# A Polypyrrole/Nanoclay Hybrid Film for Ultra-Sensitive Cardiac Troponin T Electrochemical Immunosensor

**DOI:** 10.3390/bios12070545

**Published:** 2022-07-21

**Authors:** Vicente P. A. Landim, Marcos V. Foguel, Cecília M. Prado, Maria P. T. Sotomayor, Iolanda C. Vieira, Bárbara V. M. Silva, Rosa F. Dutra

**Affiliations:** 1 Biomedical Engineering Laboratory, Department of Biomedical Engineering, Federal University of Pernambuco, Av. Prof. Moraes Rego, 1235, Recife 50670-901, Brazil; paulolandimpl@gmail.com (V.P.A.L.); mfoguel@gmail.com (M.V.F.); cica_prado@hotmail.com (C.M.P.); barbaravmsilva@hotmail.com (B.V.M.S.); 2 Institute of Chemistry, State University of São Paulo (UNESP), Araraquara 14801-970, Brazil; mpilarts@gmail.com; 3 Laboratory of Biosensors, Department of Chemistry, Federal University of Santa Catarina (UFSC), Florianopolis 88040-900, Brazil; iolanda.vieira@ufsc.br

**Keywords:** nanoclay, polypyrrole, immunosensor, cardiac troponin T

## Abstract

An electrochemical immunosensor based on a nanohybrid film of carboxylated polypyrrole and amine nanoclay was developed for label-free detection of the human cardiac troponin T (cTnT). The nanohybrid film was formed in situ on the surface of the glassy carbon electrode, followed by the covalent immobilization of anti-troponin T antibodies by glutaraldehyde. Morphological and chemical characterizations of the nanohybrid film were performed by scanning electron microscopy and Fourier-transform infrared spectroscopy. Under the optimized conditions, a calibration curve for cTnT in spiked serum was obtained by square wave voltammetry, and a low limit of detection and quantification was achieved (0.35 and 1.05 pg mL^−1^, respectively). This was the first time that this type of nanohybrid film was used in the development of an immunosensor for cTnT that proved to be a simple and efficient strategy for the manufacture of a label-free electrochemical device that could be applied in the diagnosis of acute myocardial infarction.

## 1. Introduction

The development of a sensor platform, particularly the interface between the recognition element and the transducer, has played an important role in the analytical performance of the electrochemical immunosensors and should be carefully designed to produce suitable devices. This step is crucial to improve the efficiency of the immobilization and produce sensors with high selectivity and sensitivity for the detection of analytes in low concentrations [1,2,3]. Thus, the nanoengineering of the sensor surface has been focused on promoting an increase in the electroactive area and the number of immobilized molecules, which can improve the electrical transfer and contribute to achieving better limits of detection [4]. Several nanostructured materials have been applied as support to chemical modification and biomolecules immobilization in electrochemical immunosensor, such as carbon nanotubes, graphene, nanowires, oxide/metal nanoparticles, and quantum dots [5,6,7,8,9]. A class of nanomaterials that has shown promising results is obtained from aluminosilicates, such as nanoclays (NCYs), which have been used to incorporate electroactive ions, biomolecules, and fluorescence compounds into the development of (bio)sensor devices [10,11]. NCY is a layered silicate clay mineral which consists of nanoplatelets with diameter of 50 e 200 nm in length and 1 nm in thickness [12]. Some properties of the NCYs make them suitable as electrode surface modifiers, such as their semiconductor properties, ionic exchange capacity, good catalytic support, large surface area, mechanical stability, porosity, low cost, and possibility to increase biocatalytic efficiency by reducing limitations diffusional [10,13,14].

NCY has been investigated as a nanomaterial for the development of electrochemical sensors due to its large surface area per volume, which contributes to its interaction with polymer chains to form nanocomposites [15]. The application of these nanocomposites on sensor surfaces requires the suitable adjustment of some characteristics of the film, such as thickness, porosity, and functionalization on the sensor surface. Some preparing techniques, such as Langmuir–Blodgett, layer-by-layer self-assembly, and spin coating have been used to form thin nanocomposites with NCY [16]. Another alternative is the electropolymerization of a conductive polymer on the sensor surface to form a stable and reproducible film [17]. The use of a polymer with a functional and reactive group can reduce the conductive of the polymer; however, it can facilitate the binding of the NCY on the polymeric film. Thus, due to the conductive behavior, ease of the synthesis preparation, and good stability, the polymeric film obtained from pyrrole (Py) has received great attention, especially when functionalized, allowing the use of strategic reactive groups in the modification of the sensor surface [18]. Among the functionalized monomers, Py functionalized with carboxylic acid (COOH-Py) has emerged as an electroactive material for use in biosensors [19].

This work shows, to the best of our knowledge, for the first time, the development of a label-free immunosensor based on a nanohybrid film consisting of pyrrole-2-carboxylic (COOH-Py) and aminated nanoclay (NH_2_-NCY) for the electrochemical detection of clinical levels of the cardiac troponin T (cTnT). Cardiac troponins (T and I) are highly sensitive and specific biochemical markers of myocardial cell necrosis and are widely used for the diagnosis of acute myocardial infarction (AMI). After infarction, cTnI remains detectable for 4–7 days, while the cTnT remains detectable for 10–14 days [20,21]. However, the reliable and sensitive detection of cTn in the blood just a few hours after the first symptoms can be challenging, since the peak of cTn release only occurs between 10 and 20 h after the onset of acute ischemia [22]. Thus, cTn concentrations may be underestimated, which highlights the importance to develop highly sensitive detection methodologies. Several immunoassay methods for cTnT detection have been described in the literature, including electrochemiluminescence immunoassay (ECLIA) [23], enzyme-linked immunosorbent assay (ELISA) [24], surface plasmon resonance (SPR) [25], and immune-chromatographic tests [26]. Although high-sensitivity assays for cTnT have become standard in clinical laboratories, there is still a challenge to develop a diagnostic test that combines low cost and simplicity to be applied in cardiac emergencies.

## 2. Materials and Methods

### 2.1. Reagents

Human cardiac troponin T (cTnT) antigen and mouse monoclonal anti-cTnT antibody were purchased from Calbiochem (Darmstadt, Germany). Pyrrole-2-carboxylic acid (COOH-Py) (99%), nanoclay (montmorillonite clay base material) surface modified with 0.5–5 wt.% aminopropyltriethoxysilane and 15–35 wt.% octadecylamine (NH_2_-NCY), potassium ferricyanide (K_3_[Fe(CN)_6_]), potassium ferrocyanide (K_4_[Fe(CN)_6_]), acetonitrile (anhydrous, 99.8%), glutaraldehyde (GA) (50%, *w/w*), N-hydroxysuccinimide (NHS), N-ethyl-N’-(3-dimethylaminopropyl) carbodiimide (EDC), and glycine (Gly) were obtained from Sigma-Aldrich (St. Louis, USA). Lithium perchlorate (LiClO_4_) and chloroform were purchased from Vetec (Rio de Janeiro, Brazil). Phosphate buffer saline (PBS) solution (0.01 mol L^−1^, pH 7.4) was used in all experiments for dilution of the samples. Ultrapure water was obtained from a Millipore water purification system (18 MΩ cm^−1^, Milli-Q) from Millipore (St. Louis, Missouri, USA). All the chemical reagents used to prepare buffers and solutions were of analytical grade.

### 2.2. Apparatus

Electrochemical measurements were performed by using an Ivium Compact Stat potentiostat from Ivium Technologies (Eindhoven, The Netherlands) interfaced to a microcomputer, which was controlled by the Ivium Soft TM Electrochemistry Software (version 2.419) acquired from same Ivium Technology Company. All the electrochemical measurements were performed in an electrochemical cell of 10.0 mL at room temperature, using a three-electrode system, consisting of a glassy carbon electrode (GCE) (Ø = 0.40 cm) as a working electrode, a platinum wire as an auxiliary electrode, and an Ag|AgCl|KCl_(sat.)_ electrode as a reference.

Morphology characterization and chemical composition of the nanohybrid film were performed by Scanning Electronic Microscopy (SEM) technique, using an FEI Quanta 200 FEG microscope (Eindhoven, Netherlands) equipped with energy-dispersive X-ray spectroscopy (EDX). A thin chrome layer (10 nm) was deposited by sputtering on the electrode surface. All SEM–EDX analyses were performed by using 20 kV acceleration voltages in low-vacuum mode.

Fourier-transform infrared (FTIR) spectroscopy was employed to analyze the chemical structure of the nanohybrid film. FTIR measurements in attenuated total reflectance (ATR) mode were performed by using an IFS-66 FTIR (50 scans in the range from 4000 to 400 cm^−1^ acquired from Bruker (Karlsruhe, Germany).

### 2.3. Sensor Preparation

First, the GCE surface was cleaned by polishing with alumina slurry (0.5 μm) for 3 min. Afterward, the GCE was sonicated in water and ethanol for 5 min to remove the residual alumina particles trapped on the electrode surface.

The electropolymerization of the COOH-Py on the GCE was performed by using cyclic voltammetry (CV) from −0.10 to 1.0 V at 200 mV s^−1^ for 20 successive cycles in acetonitrile containing 0.1 mol L^−1^ LiClO_4_ [27]. The carboxylic groups of the polypyrrole film were activated with EDC (2.0 mmol L^−1^) and NHS (5.0 mmol L^−1^) solutions prepared in deionized water for 60 min at room temperature. Next, 5 mg of the NH_2_-NCYs was dispersed in 1.0 mL of chloroform and sonicated for 10 min. Then 5.0 μL of the dispersed NH_2_-NCYs was drop-casted on the GCE with activated COOH-PPy film, and, subsequently, the electrode was dried for 5 min at 40 °C to evaporate the solvent. The NCY was used to increase the superficial area; thus, more antibodies can be immobilized on the electrode surface and, consequently, improve the immunosensor performance [28,29].

**Figure 1:**
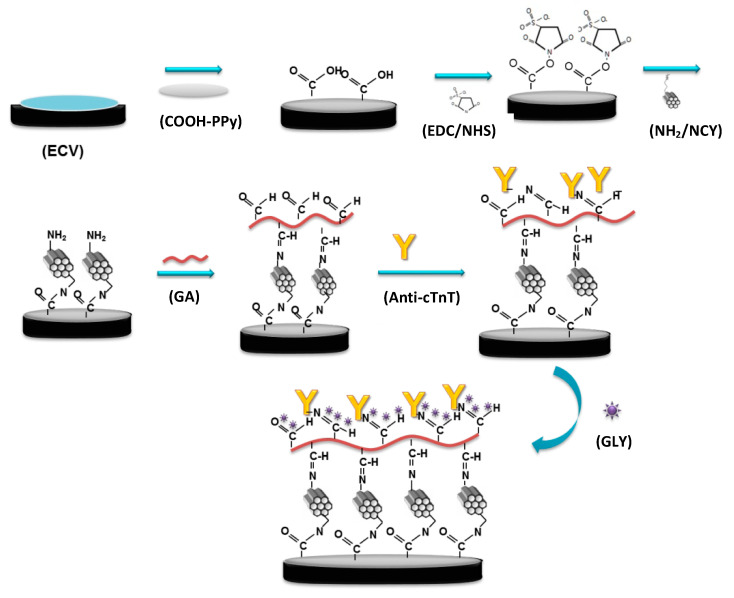
Stepwise modifications of the electrode surface for immunosensor preparation.

For the immobilization of anti-cTnT antibodies on the nanohybrid film, GA was used as a bi-functional agent. Thus, 5% GA solution was prepared in 0.01 mol L^−1^ PBS at pH 7.4, and the GCE with the nanohybrid film was immersed in this solution for 60 min. Then 10 μL of anti-cTnT were drop-casted on the GCE surface, and it was kept in a moist chamber for 60 min at room temperature. Finally, the unreacted aldehyde groups were blocked by immersing the GCE in a 0.05 mol L^−1^ Gly solution prepared in PBS (0.01 mol L^−1^, pH 7.4) for 30 min to avoid unspecific binding. All steps in the preparation of the nanocomposite on the electrode surface are represented in Figure 1.

The immunosensor was optimized and evaluated by using CV and square wave voltammetry (SWV). The CV was scanned from −0.2 to +0.7 V, at 100 mV s^−1^, and the SWV measurements were carried out from 0 to 0.5 V, at an amplitude of 10 mV, step potential of 25 mV, and frequency of 10 Hz. All electrochemical measurements were conducted in 0.1 mol L^−1^ KCl containing 5.0 mmol L^−1^ [Fe(CN)_6_]^3−/4−^.

### 2.4. Electrochemical Immunoassay

The interaction between the cTnT antigen and antibody was analyzed by incubating different concentrations of cTnT on the immunosensor for 60 min in a moist chamber, followed by washing the electrode surface with PBS (0.01 mol L^−1^, pH 7.4) to remove unbound antigens. The interaction was also monitored by SWV, using 5 mmol L^−1^ [Fe(CN)_6_]^3−/4−^ as the redox probe. The analytical response to cTnT was obtained by the difference between the peak current (ΔI) of the SWV subtracted and the blank (in absence of cTnT).

## 3. Results and Discussion

### 3.1. Characterization of the Nanohybrid Film

**Figure 2:**
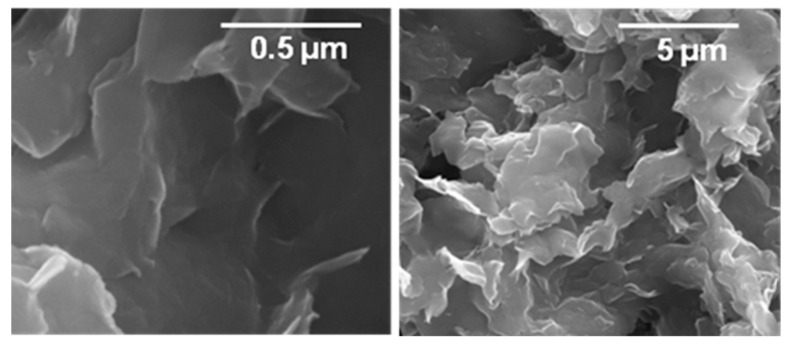
SEM images of the GCE/COOH-PPy/NH_2_-NCY at different magnifications.

The morphology of nanohybrid film (NH_2_-NCY/COOH-PPy) was analyzed by SEM images at different magnifications (Figure 2). The nanohybrid material is composed of a heterogeneous film formed by agglomerates of different two-dimensional laminar shapes as is shown in micrographies.

**Figure 3:**
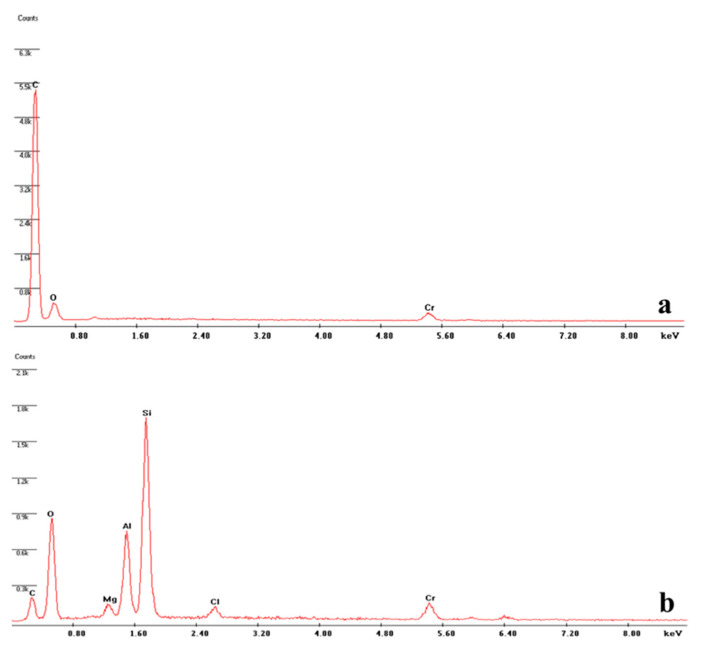
EDX spectrum of the (**a**) COOH-PPy/GCE and (**b**) NH_2_-NCY/COOH-PPy/GCE.

EDX analysis was performed to study the elementary chemical compositions of the nanohybrid film. As shown in Figure 3a, the spectrum of the GCE surface after the COOH-PPy electropolymerization has peaks corresponding to carbon and oxygen, which are the main elements of the functionalized polymer on the sensor surface. After the deposition of the NH_2_-NCY onto the polymeric film (Figure 3b), the presence of peaks referring to silicon, magnesium, and aluminum from montmorillonite nanoclay, whose chemical structure is (Na,Ca)_0.3_(Al,Mg)_2_Si_4_O_10_(OH)_2_·nH_2_O, was detected. There is also a peak corresponding to chlorine, which can be attributed to the chloroform used to disperse the nanomaterial. Meanwhile, the chromium peak in both spectra is due to the thin conductive coating applied to allow SEM analysis.

**Figure 4:**
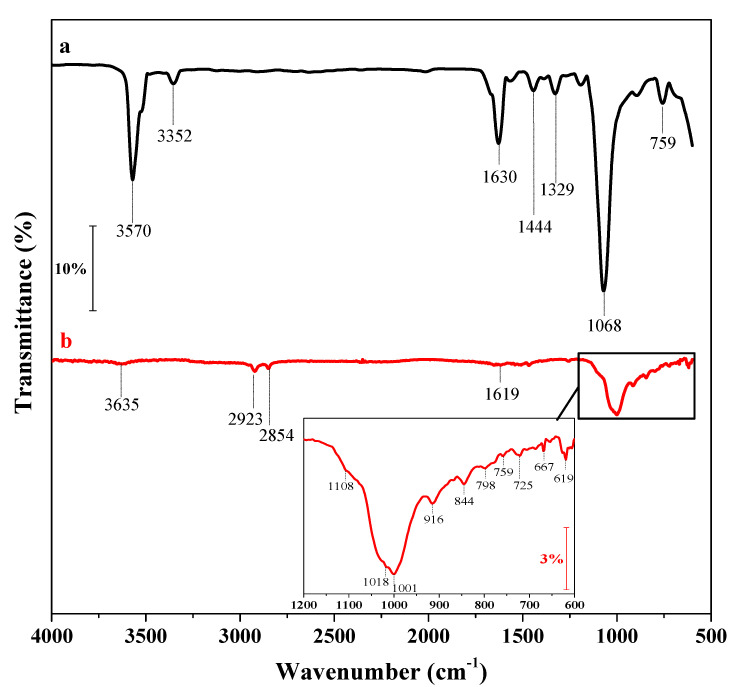
FTIR spectrum of the GCE modified with (**a**) electropolymerized COOH-PPy and (**b**) NH_2_-NCY.

Figure 4 shows the FTIR spectra of COOH-PPy and NH_2_-NCY/COOH-PPy films. The spectrum of COOH-PPy (Figure 3a) presented the characteristic bands of this compound: at 3570 cm^–1^, attributed to N–H of secondary amines of Py; at 3352 cm^–1^, corresponding to O–H stretching vibrations of the carboxylic acid group; at 1630 cm^–1^, referring to the stretching of the carbonyl group (C=O); at 1444 cm^–1^ and 1329 cm^–1^ can be attributed to pyrrolic ring stretching vibrations; at 1068 cm^–1^, the stretch corresponding to O-H out-of-plane; and at 1068 cm^–1^, the vibration of C-H out-of-plane. With the addition of NCY on the electrode surface, the spectrum (Figure 4b) showed a strong band between 1166 and 940 cm^−1^ corresponding to Si-O-Si and Si-O stretching; bands referring to Si-O also can be observed at 798 and 725 cm^−1^ due to symmetric stretching and stretching vibrations, respectively. Some weaker bands are also presented in the spectrum: at 3635 cm^−1^, attributed to OH stretching; at 2923 and 2854 cm^−1^, the bending of CH_2_ groups; at 1618 cm^−1^, corresponding to OH bending hydration; at 916 cm^−1^ and 844 cm^−1^0, referring to Al-Al-OH and Al-Mg-OH bending, respectively; and at 667 cm^–1^ and 619 cm^−1^, attributed to the vibration of OH groups of Al-OH and Mg-OH.

To evaluate the stability of the nanohybrid film, the NH_2_-NCY/COOH-PPy/GCE was submitted to 20 successive cycles voltammetric in 5 mmol L^−1^ [Fe(CN)_6_]^3−/4−^ solution. A control electrode was performed by using only the NH_2_-NCY, which was drop-casted directly onto the GCE surface (without COOH-PPy) to study the performance of the nanohybrid film. The voltammograms of the NH_2_-NCY/GCE showed a coefficient variation of the 3.2% and 2.9% for anodic and cathodic current, respectively (Supplementary Figure S1a). In comparison, the voltammograms of the NH_2_-NCY/COOH-PPy/GCE (Supplementary Figure S1b) exhibited a coefficient of variation of 0.61% and 0.44% for the anodic and cathodic peak current, respectively. These results showed that the synergism of NH_2_-NCY and COOH-PPy improves the stability of a film, which can be explained by the covalent binding between the carboxylic groups of PPy and the amino groups of the NCY formed by the EDC/NHS system.

### 3.2. Immunosensor Preparation

**Figure 5:**
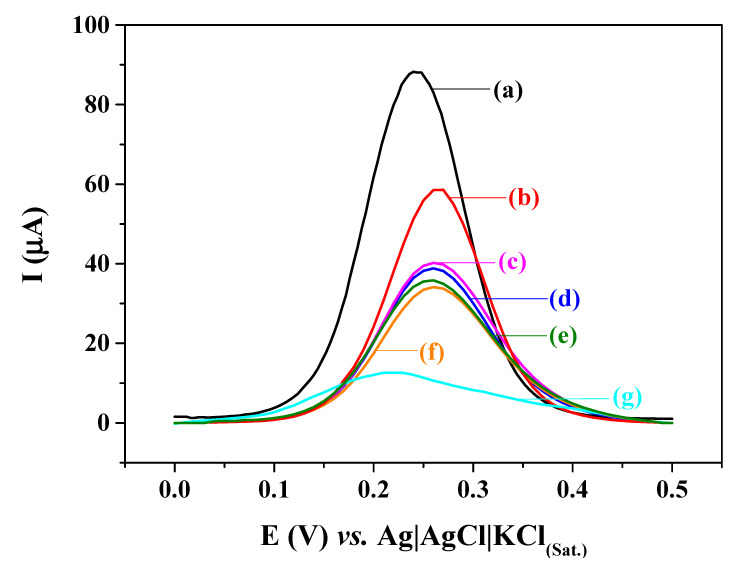
Square wave voltammograms for each step of the immunosensor assembly procedure: (**a**) bare GCE; (**b**) GCE/COOH-PPy; (**c**) GCE/COOH-PPy/NH_2_–NCY; (**d**) GCE/COOH-PPy/NH_2_–NCY/GA; (**e**) GCE/COOH-PPy/NH_2_–NCY/GA/anti-cTnT; (**f**) GCE/COOH-PPy/NH_2_–NCY/GA/anti-cTnT/Gly; and (**g**) the addition of 50 pg mL^−1^ cTnT (antigen) on the immunosensor surface. SWV measurements performed in 5 mmol L^−1^ [Fe(CN)_6_]^3−/4−^ solution prepared in 0.1 mol L^−1^ KCl.

The electrochemical characterization of each stage of the electrode preparation was performed by SWV (Figure 5). A significant decrease in redox peaks was observed between the voltammograms of the bare GCE (Figure 5a) and after electropolymerization of pyrrole (Figure 5b), evidencing that the COOH-PPy formed a layer on the electrode surface. The next modification step with NH_2_-NCY (Figure 5c) also resulted in a reduction of the faradaic current intensity, due to blockage of the electrode surface. These results showed the resistive behavior of the nanohybrid film. Afterward, GA was added to the modified electrode surface to immobilize the anti-cTnT. GA is a compound with two aldehyde groups in its structure: one of these aldehydes binds to the amino group of NCY, and the other binds to the free amines of the antibody. This reaction forms a covalent bond, and the product is an imine (Schiff base). The addition of GA on the nanohybrid film showed a slight decrease in the current intensity (Figure 5d). A reduction in current value was also observed after anti-cTnT immobilization (Figure 5e) since the antibody acts as an insulating barrier for electron transport of the redox probe. After the addition of the glycine (Gly), there was a slight reduction in the current intensity (Figure 5f). Gly was used as a deactivating agent, as it blocks aldehyde groups that do not react with anti-cTnT, which could lead to nonspecific binding on the sensor surface [30]. Finally, the immunosensor response in the presence of 50 pg mL^−1^ cTnT (antigen) resulted in a significant decrease of the peak current intensity, evidencing that the antigen can be determined by using this methodology (Figure 5g).

### 3.3. Optimization of the Experimental Conditions

The density of non-binding antibodies on the sensor surface can affect the spatial arrangement of the immobilized antibodies, which can increase the steric hindrance of antigen–antibody binding and, consequently, influence the sensibility of the methodology [31]. Thus, the influence of the amount of the anti-cTnT immobilized on the modified electrode (GCE/COOH-PPy/NH_2_-NCY/GA) was evaluated by the difference between the anodic peaks (ΔIp_a_) in the absence and presence of anti-cTnT at different concentrations (0.1, 0.5, 1.0, 2.5, 5.0, 7.5, and 10 μg mL^−1^). A plateau in the ΔIp_a_ response was obtained from 5 µg mL^−1^ anti-cTnT (Supplementary Figure S2a), and then this concentration was chosen as optimal for immunosensor development. The immunoreaction time was also evaluated at 10, 20, 40, 60, and 80 min (Supplementary Figure S2b). The maximum current intensity was observed at 60 min and then kept constant. Thus, the immunoreaction time of 60 min was chosen for further experiments.

### 3.4. Analytical Response

**Figure 6:**
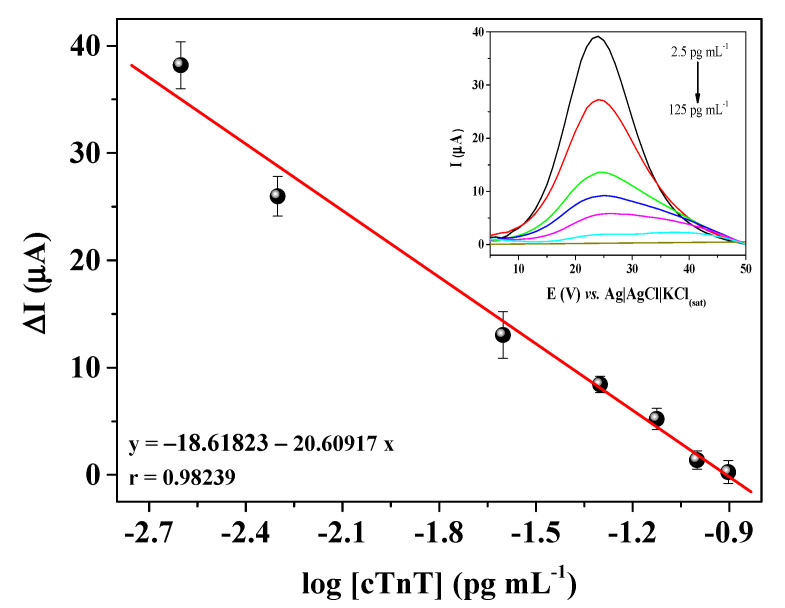
Calibration curve of the immunosensor for different cTnT concentrations (2.5, 5, 25, 50, 75, 100 and 125 pg mL^−1^) by SWV measurements performed in 5 mmol L^−1^ [Fe(CN)_6_]^3−/4−^ solution prepared in 0.1 mol L^−1^ KCl.

The calibration curve for cTnT was obtained by using a label-free assay, i.e., without a secondary antibody labeled with a redox species. The principle of the assay was based on minimizing the electron transfer kinetics of the redox probe obtained from the Faradaic process at the sensor interface, due to the insulating nature of the antigen. To build the calibration curve, the immunosensor was incubated with different concentrations (2.5, 5, 25, 50, 75, 100, and 125 pg mL^−1^) of cTnT solutions prepared in 0.01 mol L^−1^ PBS solution at a pH of 7.4. Then the immunosensor was submitted to SWV measurements in 0.1 mol L^−1^ KCl solution containing 5 mmol L^−1^ [Fe(CN)_6_]^3−/4−^. The results showed a proportional decrease of the current in relation to cTnT concentrations (Figure 6). A linear relationship was found between the immunosensor response (current) and the logarithmic of the cTnT concentration in the range from 2.5 to 125 pg mL^−1^, with the linear regression equation ΔI = −18.618 − 20.609*[cTnT], showing a correlation coefficient of 0.98239 and coefficient of variation (CV) of 7.5%. The logarithmic scale was used to linearize the calibration curve, since a concentration range with two different orders of magnitude was used. The sensibility of the immunosensor was determined by calculating the limit of detection (LOD) and the limit of quantification (LOQ) according to the IUPAC definition [32]. The LOD and LOQ of the proposed cTnT immunosensor were estimated in 0.70 pg mL^−1^ and 2.10 pg mL^−1^, respectively.

### 3.5. Immunosensor Response to Serum Samples

**Figure 7:**
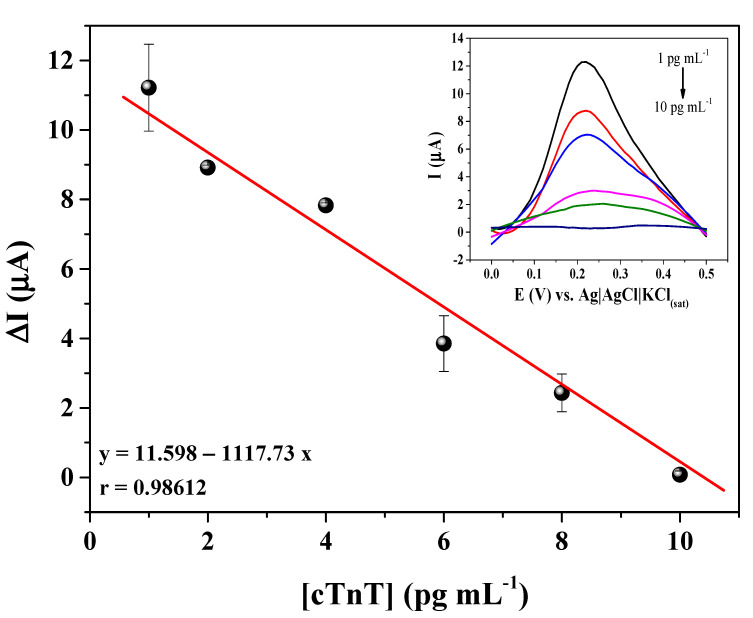
Calibration curve of the immunosensor in a spiked serum sample for different cTnT concentrations (1, 2, 4, 6, 8, and 10 pg mL^−1^) measurements performed in 5 mmol L^−1^ [Fe(CN)_6_]^3−/4−^ solution prepared in 0.1 mol L^−1^ KCl.

First, the serum dilution was analyzed by comparing the current intensity between serum in the absence and presence of cTnT. In this study, the serum was spiked with 50 pg mL^−1^ cTnT. The following dilutions were studied: 1:50; 1:25; 1:12,25; 1:8,33; 1:6,25; and 1:5. The results are shown in Supplementary Figure S3. All studied dilutions presented a significant difference between serum with and without cTnT, with current intensity about 2-fold higher for the spiked sample, which shows that the immunosensor response was not affected by the serum matrix. The 1:5 dilution can be highlighted because the difference was slightly bigger (2.2-fold), and the current intensity was higher than the other dilutions, which improves the sensibility of the immunosensor. Thus, the calibration curve in spiked serum was performed by using a dilution of 1:5. Then a serum without cTnT was contaminated with cTnT in the concentration range of 1.0–10 pg mL^−1^ (Figure 7). The immunosensor presented a linear response in the studied concentration range, with a linear regression equation equal to ΔI = 11.598 − 1117,73*[cTnT] and a correlation coefficient of 0.98612. The LOD and LOQ of the proposed cTnT immunosensor in a serum sample were estimated to be 0.35 and 1.05 pg mL^−1^, respectively. These limits are lower than the clinical levels, which may contribute to the early diagnosis of cTnT in patients after the first hours of infarction symptoms.

## 4. Conclusions

The nanohybrid film of the COOH-PPy and NCY showed to be an efficient platform to provide a covalent binding of the antibodies by the reaction with glutaraldehyde. The proposed label-free electrochemical immunosensor for troponin T, using SWV, demonstrated satisfactory properties, such as good sensitivity and low limit detection. The cTnT concentration that the immunosensor may detect is equivalent to clinical levels for the diagnosis of acute myocardial infarction. Therefore, this electrochemical immunosensor presents as a promising alternative to be used in cardiac emergencies.

## Supplementary Materials

The following are available online at https://www.mdpi.com/article/10.3390/bios12070545/s1. Figure S1: Electrochemical stability of the modified GCE with NH_2_-NCY film and NH_2_-NCY and COOH-PPy film. Figure S2: Influence of the anti-cTnT concentration and incubation time of the cTnT. Figure S3: Current intensity response of the immunosensor for different serum dilutions.
