# Supplementary material for: A Polypyrrole/Nanoclay Hybrid Film for Ultra-Sensitive Cardiac Troponin T Electrochemical Immunosensor
Source: Biosensors (Basel). 2022 Jul 21;12(7):545. doi: 10.3390/bios12070545 (PMC9313248; doi:10.3390/bios12070545)
Supplement: Supplementary file 1 [file biosensors-12-00545-s001.zip › biosensors-1768905-supplementary.pdf]

SUPPORT INFORMATION

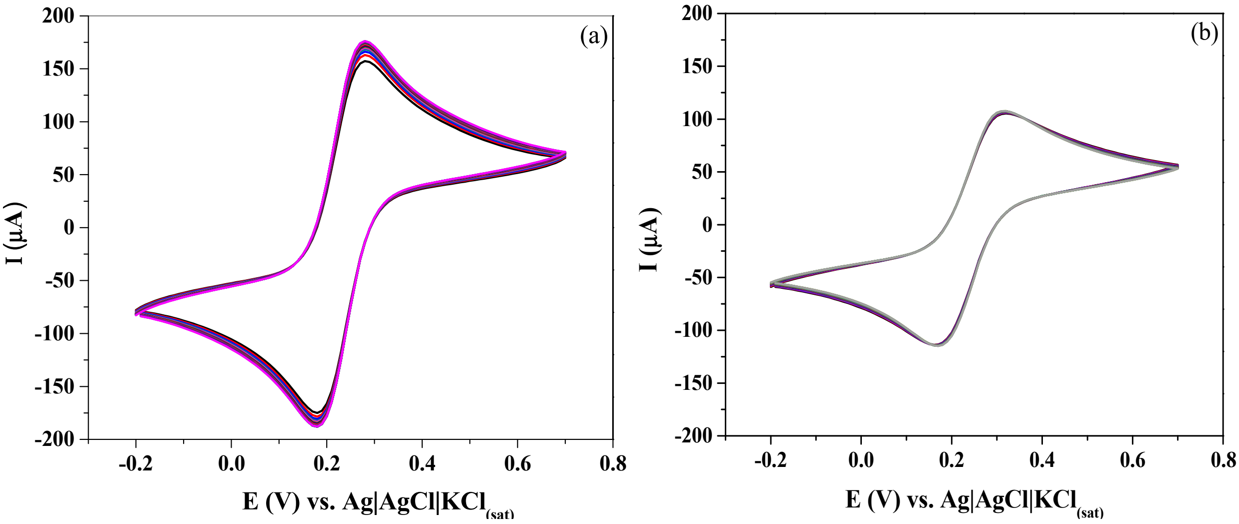

Figure S1. Electrochemical stability of the modified GCE with NH_2-NCY film (a) and NH_2-NCY and COOH-PPy film (b) when submitted to 20 successive voltammetric cycles. Measurements performed in 5 mmol L^{-1} $K_3Fe(CN)_6/K_4Fe(CN)_6$ prepared in 0.1 mol L^{-1} KCl.

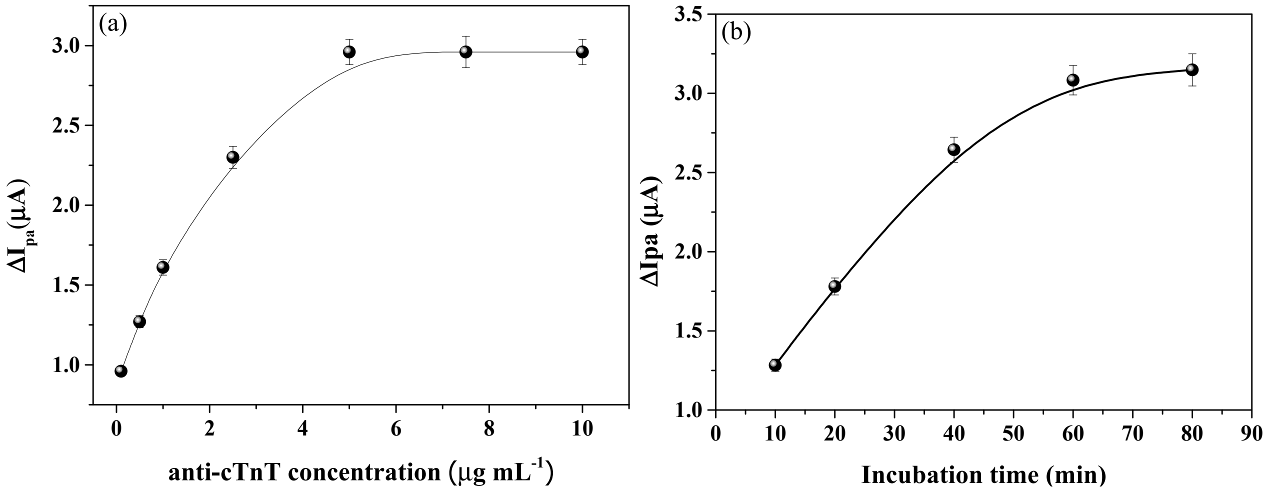

Figure S2. Influence of the anti-cTnT concentration (a) and incubation time of the cTnT (b) on the anodic current response by SWV measurements performed in 5 mmol L^{-1} $[Fe(CN)_6]^{3-/4-}$ solution prepared in 0.1 mol L^{-1} KCl.

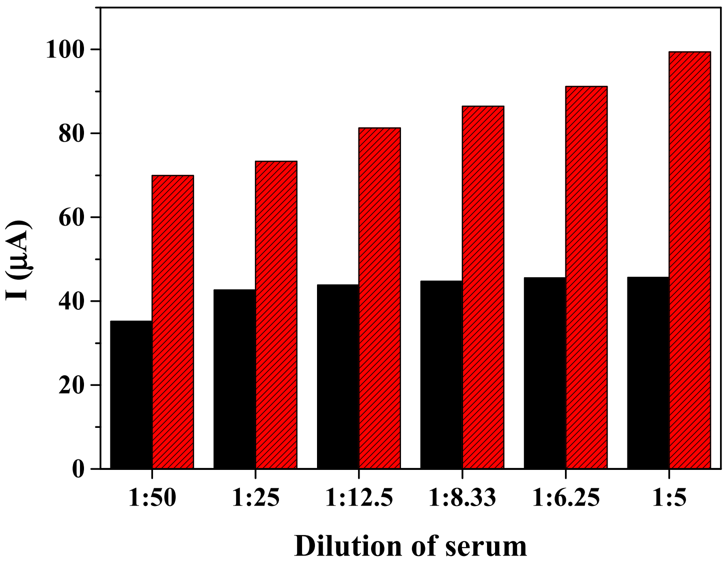

Figure S3. Current intensity response of the immunosensor for different serum dilutions (1:50; 1:25; 1:12,25; 1:8,33; 1:6,25; 1:5) by SWV measurements performed in 5 mmol L^{-1} $[Fe(CN)_6]^{3-/4-}$ solution prepared in 0.1 mol L^{-1} KCl. Black bars represent the serum in absence of cTnT and the red bars represent the serum spiked with 50 pg mL^{-1} cTnT.
